# Monitoring Electrical Biasing of Pb(Zr_0.2_Ti_0.8_)O_3_ Ferroelectric Thin Films In Situ by DPC-STEM Imaging

**DOI:** 10.3390/ma14164749

**Published:** 2021-08-23

**Authors:** Alexander Vogel, Martin F. Sarott, Marco Campanini, Morgan Trassin, Marta D. Rossell

**Affiliations:** 1 Electron Microscopy Center, Empa, Swiss Federal Laboratories for Material Science and Technology, 8600 Dübendorf, Switzerland; marco.campanini1@gmail.com; 2 Department of Materials, Eidgenössische Technische Hochschule Zürich, 8093 Zürich, Switzerland; martin.sarott@mat.ethz.ch (M.F.S.); morgan.trassin@mat.ethz.ch (M.T.)

**Keywords:** in situ TEM, differential phase contrast, ferroelectric, thin film capacitor, PZT, electrical biasing

## Abstract

Increased data storage densities are required for the next generation of nonvolatile random access memories and data storage devices based on ferroelectric materials. Yet, with intensified miniaturization, these devices face a loss of their ferroelectric properties. Therefore, a full microscopic understanding of the impact of the nanoscale defects on the ferroelectric switching dynamics is crucial. However, collecting real-time data at the atomic and nanoscale remains very challenging. In this work, we explore the ferroelectric response of a Pb(Zr_0.2_Ti_0.8_)O_3_ thin film ferroelectric capacitor to electrical biasing in situ in the transmission electron microscope. Using a combination of high-angle annular dark-field scanning transmission electron microscopy (HAADF-STEM) and differential phase contrast (DPC)-STEM imaging we unveil the structural and polarization state of the ferroelectric thin film, integrated into a capacitor architecture, before and during biasing. Thus, we can correlate real-time changes in the DPC signal with the presence of misfit dislocations and ferroelastic domains. A reduction in the domain wall velocity of 24% is measured in defective regions of the film when compared to predominantly defect-free regions.

## 1. Introduction

Ferroelectrics have found a large number of applications in modern life since their discovery a century ago [1]. Their auxiliary properties, such as piezoelectricity and pyroelectricity, have led to the development of actuators, sensors and dielectric capacitors based on ferroelectric materials that outperform their non-ferroelectric competitors. In data storage applications, ferroelectricity presents a unique alternative to ferromagnetism as it allows for more energy efficient and faster devices [2]. However, the commercialization of ferroelectric random-access memory (FeRAM) chips has been restricted to electronic devices storing small amounts of data. Further miniaturization of ferroelectric-based memories is hindered by major reliability issues [2,3] such as retention loss [4], imprint [5] and fatigue [6]. These are typically related to the presence of structural and charged defects, which affect the domain wall motion and domain nucleation [7,8,9]. Yet, at present, our understanding of the switching processes involved in ferroelectric systems is largely impeded by insufficient dynamical data on the atomic and nano-scale.

Over the last decade, major insights into the nanoscale processes involved in ferroelectric switching were driven by the development of specialized transmission electron microscopy (TEM) systems for in situ experiments using a conductive nano-sized probe brought into contact with the specimen using a piezo-micromanipulator [8,10,11,12,13,14,15]. The importance of interfaces and their associated defects were confirmed by combining these holders with atomic resolution scanning TEM (STEM) imaging as well as conventional dark-field TEM [10,11]. It was further shown that ferroelastic domains can be permanently stabilized by dislocations at the substrate interface, while similar domains without such pinning dislocations can behave more freely [8]. More recent developments demonstrated deterministic ferroelastic domain switching in an epitaxial bilayer system by in situ electrical biasing in the TEM [14]. Nevertheless, the probe-based in situ biasing experiments have a few disadvantages. First, the electric fields underneath the needle-like tip are strongly inhomogeneous, making their quantification challenging. Second, the strain induced by the force of the needle pushing against the film might cause piezoelectric and ferroelastic responses of the studied material. A third, more practical disadvantage is the limited availability of these specialized TEM in situ biasing systems, as well as the concomitant time-consuming and demanding sample preparation procedures. One approach to overcome the first two issues encompasses the introduction of a top-electrode deposited during the sample preparation, which is then contacted far away from the region of interest in the film [11]. Instead, we have developed a workflow to prepare ferroelectric thin film specimens on commercially available microelectromechanical systems (MEMS) half-chips using a focused ion beam (FIB) allowing more accessible in situ electrical biasing experiments. Our approach permits the application of a homogeneous electric field across the probed specimen area.

Various ferroelectric systems have been studied over the last decades, ranging from the common perovskite ferroelectrics, such as BaTiO_3_ [16,17,18,19], multiferroic materials [20,21], such as BiFeO_3_ [22,23,24] and hexagonal manganites [25,26], multilayered structures of different ferroelectrics [14,27,28] to ferroelectric polymers [29,30,31,32]. In the prototypical perovskite ferroelectrics, the spontaneous polarization originates from the off-center displacement of the transition metal cation at the center of the oxygen octahedron. Accordingly, domain boundaries in these systems can act both as ferroelastic and ferroelectric walls and result in a strong coupling of the electromechanical material properties. In tetragonal bulk crystals, this displacement occurs along one of the three principal axes of the crystal lattice. Once the structural distortion is established, the spontaneous polarization can take one of two energetically equivalent states, amounting to six possible domain states. In the context of perovskite ferroelectric thin films, one further differentiates between out-of-plane polarized domains (*c*-domains) and in-plane polarized domains (*a*-domains). The distribution of these domains and their associated domain walls are of high importance for the technological merit of the ferroelectric thin films. For example, the presence of *a*-domains plays a critical role in the formation of flux-closure domain patterns [33]. Then again, *a*-domains, originating from surface or interface dislocations, lead to immobile 90° domain walls between *a*- and *c*-domains, obstructing the mobility of 180° domain walls [7,8].

In particular, the Pb(Zr_x_Ti_1−x_)O_3_ (PZT) system is the technologically most important ferroelectric, mostly because of its high piezoelectric response [34]. Accordingly, a large number of studies on the PZT system have been conducted, covering the full compositional spectrum and spanning from the macroscopic to microscopic length scales [3,34,35,36,37,38,39,40,41,42]. These studies address many topics. In the context of the ferroelectric switching process, they comprise the confirmation of conductive domain walls in PZT [43,44], first discovered in the multiferroic BiFeO_3_ [24], and plenty of studies on the domain dynamics employing different experimental techniques. Scanning probe microscopy (SPM) studies showed that the domain wall motion can be described as a creep process [45] and confirmed the inhomogeneous nature of domain nucleation in ferroelectrics [46]. Others focused on combining SPM techniques with synchrotron X-ray diffraction (XRD) to investigate the substrate clamping effect on the piezoelectric response in PZT [47]. Yet others employed TEM-based techniques to investigate the strain-induced ferroelastic properties [48] and the coupling between the ferroelastic and ferroelectric properties down to the atomic scale [8,13]. Some studies have also demonstrated the possibility to overcome the substrate clamping effects, assumed to strongly pin ferroelastic domains and impede domain wall motion, by manufacturing multilayered structures [49]. Still, a complete understanding of the detailed interactions between the material microstructure and both ferroelectric and ferroelastic domains has not yet been achieved [50,51,52].

In this work, we report on in situ electrical biasing investigations of ferroelectric Pb(Zr_0.2_Ti_0.8_)O_3_ thin films, which have one of the highest spontaneous polarization of the lead zirconate-titanate Pb(Zr_x_Ti_1−x_)O_3_ solid solution [53], integrated into a capacitor architecture. A first complete structural characterization of the thin film and its polarization state is performed by combining different STEM techniques, followed by an analysis of the response of the PZT film to electrical bias inside the TEM. The local dynamics of the switching process are correlated with the underlying crystal structure and the role of defects. Thus, the reduction in the velocity of the domain wall motion caused by the presence of misfit dislocations and *a*-domains is quantitatively evaluated in the technology relevant capacitor design. 

## 2. Materials and Methods

### 2.1. Thin Film Deposition

The thin film heterostructures were grown on single crystalline 0.1% Nb-doped SrTiO_3_ (001) (Nb:STO) with pulsed laser deposition (PLD) using a 248 nm KrF excimer laser. An additional (001)-oriented SrRuO_3_ epitaxial buffer 2 unit-cell thick was deposited at a substrate temperature of 700 °C with a laser fluence of 0.95 J/cm^2^ at a repetition rate of 2 Hz and an oxygen growth pressure of 0.1 mbar. The (001)-oriented Pb(Zr_0.2_Ti_0.8_)O_3_ layer was grown at 550 °C with a laser fluence of 1.2 J/cm^2^ at 2 Hz and a growth pressure of 0.12 mbar. After growth, the oxygen pressure was increased to 200 mbar and the films were cooled at 10 K/min to room temperature. The pristine ferroelectric domain state of the film was investigated by piezoresponse force microscopy (PFM). ±4 V was reversibly applied to the scanning tip to locally switch the out-of-plane oriented polarization of the film, see Supplementary Figure S1. The film exhibits a root-mean-square roughness of 0.26 nm.

### 2.2. Scanning Transmission Electron Microscopy

**Figure 1:**
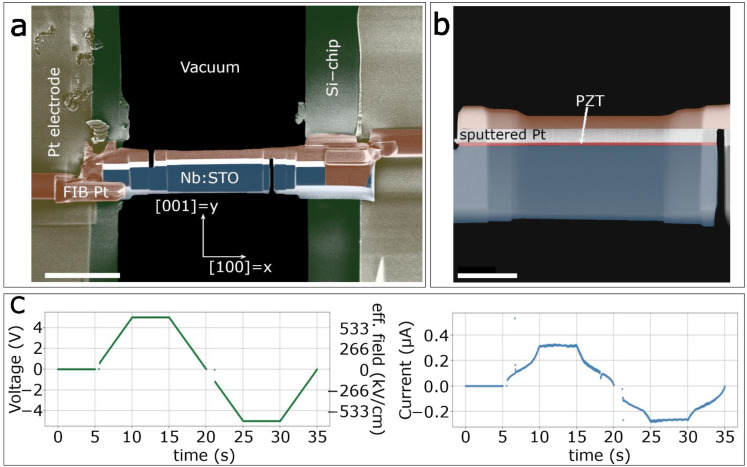
(**a**) False colored scanning electron microscopy (SEM) image and (**b**) false colored high-angle annular dark-field scanning transmission electron microscopy (HAADF-STEM) image of a lamella for in situ biasing prepared by focused ion beam (FIB). Yellow: Pt electrodes of the microelectromechanical systems (MEMS) chip. Orange: FIB-deposited Pt. Green: Si-MEMS chip, Blue: Nb-doped SrTiO_3_ (Nb:STO) substrate. Red: Pb(Zr_0.2_Ti_0.8_)O_3_ (PZT) thin film. The scale bars are 5 µm and 2 µm, respectively. (**c**) Applied voltage and measured current profiles during the in situ biasing experiments.

The PLD grown thin films are capped with a 400 nm thick Pt layer using sputtering. The Pt layer acts both as top electrode and protective layer during FIB sample preparation. Cross-sectional specimens for in situ biasing experiments were then prepared on MEMS half-chips designed by Protochips using a FEI Helios Nanolab 660 FIB/SEM operated at acceleration voltages of 30 and 5 kV. A false colored SEM image is shown in Figure 1a. Here, yellow shaded regions are the pre-manufactured Pt electrodes of the MEMS chip, which are connected to the lamella by FIB deposited Pt, colored in orange. The conductive Nb:STO substrate (blue) acts as the bottom electrode. Two FIB cuts through the top and bottom electrodes, respectively, prevent short-circuiting. The crystallographic orientation is indicated at the bottom of Figure 1a, and in the following we will refer to the [100] and [001] directions as x- and y-directions, respectively. The final thinning steps are performed at an acceleration voltage of 5 kV and a beam current of 41 pA. In these steps, the entire lamella was cleaned of any metal halos that may have formed due to redeposition of milled material. In this way, the chance of short-circuiting through metal halos is reduced. The final lamella is shown in the false colored high-angle annular dark-field (HAADF)-STEM micrograph in Figure 1b, with the PZT thin film highlighted in red. A FEI Titan Themis equipped with a probe CEOS DCOR spherical aberration corrector operated at 300 kV is used for both HAADF- and differential phase contrast (DPC)-STEM imaging. A probe semi-convergence angle of 17 mrad was set in both imaging modes, with an annular semi-detection range of 66–200 and 9–57 mrad for the annular dark-field and segmented annular detector, respectively. 

### 2.3. In Situ Electrical Biasing

We perform in situ electrical biasing experiments using a Protochips Fusion Select holder for in situ TEM biasing and a Keithley Model 2636B, controlled by the Protochips Clarity Software, which acts as a voltage source. The conductive Nb:STO substrate is kept at 0 V, while the voltage is applied to the Pt top electrode. The voltage profile consists of an initial 5 s interval without applied voltage to record the initial state of the PZT film before biasing, followed by a continuous ramp of 1 V/s up to a maximum positive voltage of 5 V. The maximum voltage is held for 5 s before another continuous ramp of 1 V/s is applied until a maximum negative voltage of −5 V is reached, which is again held for 5 s before returning to 0 V with a final 1 V/s ramp. The voltage profile and measured current during the experiment are shown in Figure 1c. Additionally, the effective electric field in kV/cm is also given to facilitate comparison with other reported biasing studies. The DPC signal was recorded during the electrical biasing with a dwell time of 400 ns and a resolution of 1024 × 1024 pixels, resulting in a scan time of about 420 ms per frame.

### 2.4. Data Processing and Analysis

Averaged HAADF-STEM images were obtained after non-rigid registration of time series of 10 frames using the SmartAlign software [54]. Geometric Phase Analysis (GPA) was performed using the FRWRtools [55] plugin for Digital Micrograph, following the theoretical framework by Hÿtch et al. [56]. Atomic column fitting is performed using the python library atomap [57] after applying a custom-developed python-based probe deconvolution algorithm on the averaged HAADF-STEM images. 

**Figure 2:**
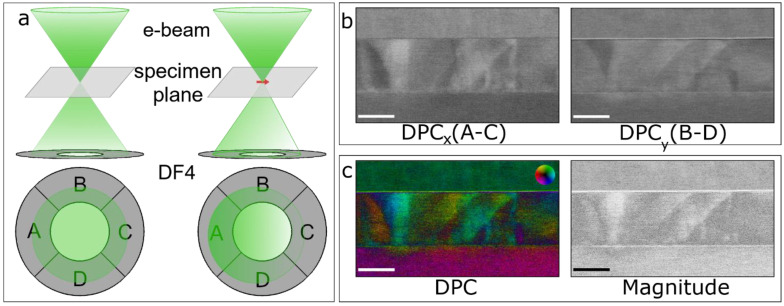
(**a**) Schematic representation of the basic principle of differential phase contrast (DPC)-STEM imaging. (**Left**) Electrons passing through vacuum. (**Right**) The electron beam is deflected by an electric field represented by the red ar-row. (**b**) Differential signals between two opposing detector segments (A − C, B − D). (**c**) Vector map of the DPC signal (**left**) and magnitude of the DPC signal (**right**). The scale bars are 50 nm.

Figure 2a shows a schematic illustration of the medium-resolution DPC-STEM imaging technique. On the left, the convergent electron probe is shown passing through vacuum. In this case, the undisturbed transmitted disk homogeneously illuminates all 4 detector segments. The right side illustrates the effect of an electric field caused by the electrostatic potentials present in the specimen plane, which are represented by a small red arrow. Here, the electron probe is deflected by the electric field, resulting in a shift of the center of mass of the transmitted disk. The DPC images are then determined from the 4 detector segments following the description by Lazic et al. [58]. First, the difference images are calculated from opposing segments of the detector, i.e., (A − C) and (B − D), as seen in Figure 2b. These differential signals are then normalized using the sum of all four detector segments, i.e., (A − C)/(A + B + C + D), which reduces the contrast variation due to absorption effects. The electric field is then computed as I=M∗exp(i∗θ), where θ=arctan([A−C]/[B−D]) and $M\;=\sqrt{{\left(A-C\right)}^{2}+{\left(B-D\right)}^{2}\;}$ are the phase and magnitude of the signal, respectively. A vector plot of this signal is shown on the left side of Figure 2c, where the color of the image corresponds to the phase of the signal and the saturation corresponds to the magnitude. The different colors present in the DPC image are caused by minor misalignments from the zone-axis of the PZT film due to the presence of structural defects, as it will be discussed later. For comparison, the calculated magnitude of this signal without any phase information is shown on the right side of Figure 2c.

## 3. Results and Discussion

### 3.1. Characterization of the As-Grown State

**Figure 3:**
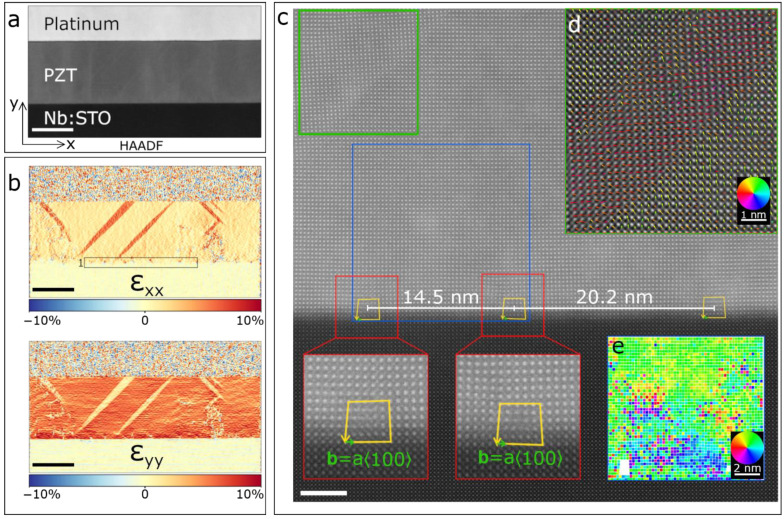
(**a**) HAADF-STEM image of the PZT thin film grown on Nb:STO and Pt electrode. (**b**) Relative strain maps along x- and y-directions calculated by geometric phase analysis (GPA) of the HAADF-STEM image shown in (**a**). The substrate was used as zero strain reference. Area 1 highlights a row of misfit dislocations at the PZT/Nb:STO interface. (**c**) Atomic resolution HAADF-STEM image showing a row of dislocations at the interface and a section of a needle-like domain at the top left corner. Magnified views of the dislocations with the Burgers circuits and resulting Burgers vector are shown at the bottom. (**d**) Polarization map superimposed to the ferroelastic needle-like domain showing the polarization rotation, calculated from the atomic displacements of the Ti ions with respect to the 4 neighboring Pb ions. The color and size of the arrows correspond to the direction and magnitude of the polarization, respectively. (**e**) Map of the polarization direction of the region comprising the two dislocations on the left side (blue rectangle). A region near the bottom interface with reversed (downwards) polarization is clearly observed in blue color. Scale bars in (**a**,**b**) are 50 nm and in (**c**) 5 nm, respectively.

The initial state of the PZT film before biasing is summarized in Figure 3. Figure 3a shows a HAADF-STEM image of a 75 nm thick PZT film inserted in a vertical capacitor geometry with the Pt top electrode and the conductive Nb:STO substrate. We then map in Figure 3b the strain of the PZT film relative to the Nb:STO substrate using GPA. In the top part of Figure 3b the in-plane strain ε_xx_ is plotted, showing an average in-plane lattice expansion of 2% in the mainly *c*-axis oriented PZT film with respect to the cubic substrate. Needle-like *a*-domains originate both from the bottom interface (and typically propagate through the entire film thickness) and from the middle of the PZT film. In these *a*-domains the in-plane lattice elongation reaches about 5%, confirming the 90° rotation of the tetragonal lattice. In the bottom part of Figure 3b, a similar behavior is observed, with the out-of-plane strain ε_yy_ being 5% and 2% larger for the *c*- and *a*-domains, respectively. In Figure 3c, an atomically resolved HAADF-STEM image of a section of the film is shown. The PZT lattice parameters, measured using the STO lattice parameter $a_{STO}=3.905$ Å as an internal reference, are a=3.975 Å and c=4.120 Å in the in-plane and out-of-plane directions, respectively. Despite the nominally moderate compressive lattice strain imposed by the substrate, a closer look at the evolution of the PZT lattice parameters from the bottom interface to the PZT film surface, shown in Supplementary Figure S2, reveals a relaxation of the in-plane a parameter to bulk-like values from 3.905 Å to 4.02 Å within the first 5 unit cells, while a relaxation towards 3.975 Å follows in the next 16 atomic rows. A slight difference from the values found in bulk PZT ceramics is expected here because of the epitaxial strain imposed by the substrate and interfacial defect formation [59]. The ~11.5% expansion of the in-plane parameter across only 5 unit cells is allowed by the presence of quasi-periodic misfit dislocations at the STO/PZT interface, which are also identified in Figure 3b and marked as area 1. Figure 3c depicts a representative HAADF-STEM image of three edge misfit dislocations separated by 14.5 and 20.2 nm, respectively. In the magnified views shown at the bottom of the figure, yellow arrows show the Burgers circuits and the resulting Burgers vectors (marked by green arrows) are b=a⟨100⟩ for all three dislocations. In addition, at the top left corner of the image a section of an *a*-domain needle is also imaged. This structural analysis will later allow us to correlate changes in the DPC signal during in situ biasing with the underlying thin film crystal structure. 

Let us now shift our focus on the initial polarization state of the film. The inset in Figure 3d shows the resultant polarization map created by mapping the atomic displacement of the Ti ion with respect to its 4 neighboring Pb ions after atomic column fitting. Here, the color of the arrows corresponds to the direction of the polarization as indicated by the color wheel. The film is seen to be mainly upwards polarized, while the *a*-domain shows the characteristic 90° rotation of the polarization. Furthermore, Figure 3e displays the polarization map extracted from the region located between the two dislocations on the left side (blue rectangle). In this region, up to about a thickness of 8 nm, the polarization is reversed (downwards) compared to the rest of the film. As a result, energetically unfavorable charged domain walls with tail-to-tail configuration are established in the PZT film. Oxygen vacancies have been shown to accumulate in this type of domain walls and to act as effective pinning centers to the domain wall motion [60,61,62].

### 3.2. In Situ Electrical Biasing

With the as-grown state fully characterized, we move on to the in situ biasing experiments. The movie in the supplementary material SV1 shows the DPC signal of the full biasing experiment. We start with a complex domain state of primarily out-of-plane polarized domains, with contributions of the interfaces and needle-like domains. This mixed state with upwards and downwards polarization domains is comparable to the non-switchable PZT domains previously reported by Han et al. [11]. At 5 s, we start increasing the voltage by 1 V/s. After 9 s have passed, at about 4 V, the DPC signal begins to change and we can see a front getting pushed down throughout the film, causing a color shift to more homogeneous green, indicating a fully upwards polarization. This rapid switching takes place in only about 1.9 s, with the biggest change occurring between 4.6 and 5.0 V. No additional changes are observed during the next 5 s, when the film is kept at 5.0 V. During negative switching, a voltage rate of −1 V/s is applied. At roughly 0.8 V, a 180° switching front, represented by a dark front, begins to emerge from the bottom interface of the film and we observe the 180° domain wall motion as it traverses the film towards the top within approximately 2.6 s. This process ends at −1.8 V and no further modifications are observed in the film. Finally, the PZT film returns to its initial state, with the formation of opposite polarization domains in a tail-to-tail configuration, as the voltage is increased back to 0 V. This polarization backswitching, occurring on the removal of the external field, is known to induce retention loss in ferroelectric thin films and is caused by built-in fields originating from the different work functions of Nb:STO and Pt [59,63,64]. In our samples, backswitching is mainly localized in the first ~8 nm of the PZT film near the bottom interface and results in charged domain walls with tail-to-tail configuration, as shown in Figure 3e. These charged domain walls are stabilized by mobile charge carriers in the PZT film [11].

**Figure 4:**
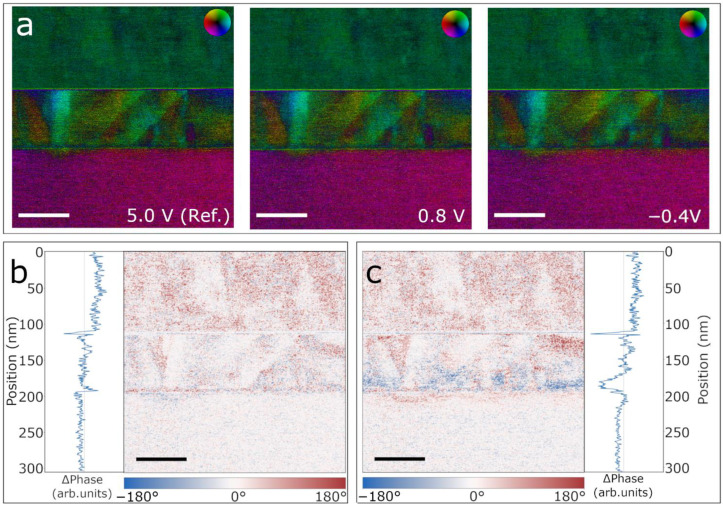
(**a**) DPC vector color plots of the electric field of the PZT film at 5 V (used as a reference), 0.8 V and −0.4 V, where the hue gives the field direction and the saturation is proportional to the vector modulus. Note the difficulty of detecting the changes in the polarization state of the film in the DPC vector plots. (**b**) Phase difference map obtained by subtracting the 5 V and 0.8 V DPC vector plots. (**c**) Phase difference map obtained by subtracting the 5 V and −0.4 V vector plots. The line profiles of the difference maps are extracted over the full image widths. The scale bars are 50 nm.

Following this process in the DPC signal is very challenging as minor sample misalignments result in significant color changes, as previously pointed out and shown in Figure 2c. The three DPC images shown in Figure 4a exemplify the difficulty of observing the dynamic changes during the biasing experiment from individual frames shown side-by-side. A way to overcome this problem is to track the evolution of the switching front by calculating the difference between two images acquired at different moments of the biasing experiment. In Figure 4, we describe this procedure by taking three DPC images acquired during the negative switching. In particular, as shown in Figure 4a, we use the DPC signals at 5.0 V (here the switching front is completely pushed down to the bottom interface and the film is fully upwards polarized), 0.8 V (the front has not yet started moving towards the top interface) and −0.4 V (the front is located roughly at the middle of the PZT film). The signal acquired at 5.0 V (initial state) is used as reference. The obtained difference images are shown in Figure 4b (as the difference in phase between 5.0 V and 0.8 V) and Figure 4c (difference between 5.0 V and −0.4 V). The blue color in the difference maps relates to a phase change of up to −180° and the red color of up to +180°, while the line profiles on the side are obtained by averaging the phase change along the horizontal direction. Of particular interest in these results is the blue front that has clearly developed from the Nb:STO/PZT interface in Figure 4c and is not yet present in Figure 4b. A negative phase change at the 180 nm mark in the line profile shown in Figure 4c, not observed in Figure 4b, further illustrates this difference.

**Figure 5:**
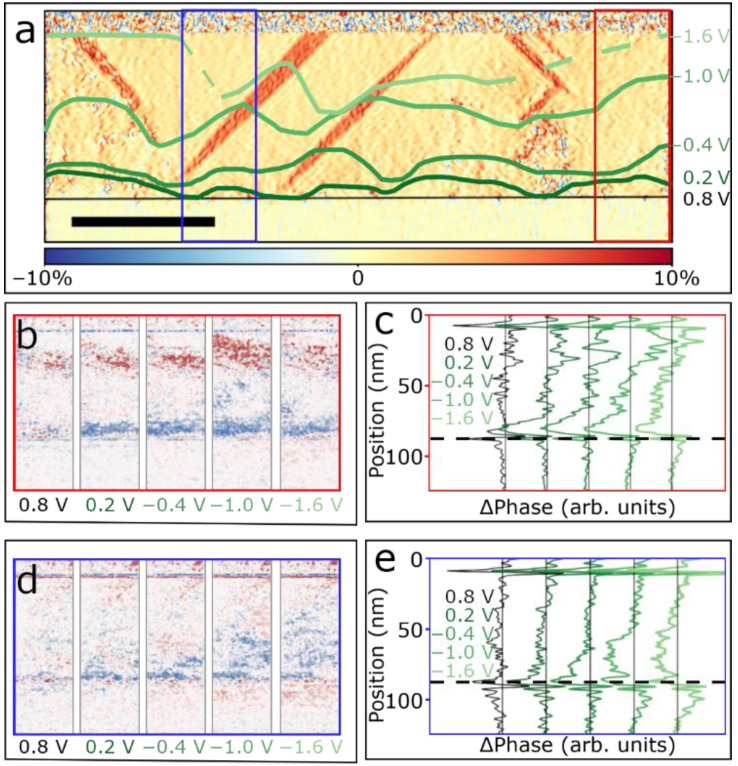
(**a**) In-plane ε_xx_ strain map with an overlay of the estimated position of the switching front throughout the experiment. The red and blue rectangles correspond to the regions shown in (**b**,**d**), respectively. The scale bar is 50 nm. (**b**,**d**) Slices of the phase change plots at different voltages, showing the progression of the switching front in a predominantly defect free region (**b**) and a region enclosing a ferroelastic needle-like domain (**d**). (**c**,**e**) Line profiles of the average phase change extracted along horizontal direction in the regions shown in (**b**,**d**), respectively.

In Figure 5 we follow the evolution of the polarization from fully upwards to downwards between 0.8 V and −1.6 V in more detail. In Figure 5a, the switching front extracted from the phase difference at various voltages (as explained in Figure 4) is superimposed on the ε_xx_ GPA strain map allowing us to correlate the presence of defects with the motion of the switching front. In the early switching stages, at 0.2 V, the front starts emerging at the bottom interface, extending into the film at different speeds. Near the leftmost and rightmost edges of the frame, i.e., regions with lower defect density, the front moves fastest, while in the center region, where both needle-like domains and misfit dislocations are present, the front is pinned at some of the defects. Thus, domain switching is hindered by structural defects and the switching front displays a wavy-like shape. This trend continues with decreasing voltage and the front gets stuck at the boundaries of the needle-like domains until, at a bias of approximately −1.6 V, it overcomes the ferroelastic domains and proceeds all the way through the rest of the film. A similar behavior was previously observed in PZT thin films switched in situ making use of a conductive nanoprobe [7]. For the applied voltages in this study, no annihilation of the ferroelastic domains was ever observed [52].

To extract quantitative data out of our biasing experiments we identified two film regions, which are indicated by a red and blue rectangle in Figure 5a. The region marked with a red rectangle corresponds to a predominantly defect-free area (though two misfit dislocations are present at the bottom interface), while the region marked with a blue rectangle is representative of a highly defective region, comprising a needle-like domain in addition to misfit dislocations at the bottom interface. These regions are further analyzed in Figure 5b–e, again using the phase difference to visualize the dynamic changes. Particularly, Figure 5b–d display the phase difference maps of the two regions, while Figure 5c–e show the phase difference at various voltages averaged along the horizontal direction. In the predominantly defect-free area of Figure 5b we can follow a negative phase difference front, seen with blue color, emerging from the bottom interface. This front moves homogeneously across the film from 0.8 to −0.4 V. From −1.0 to −1.6 V the motion of the front becomes less clear because of the positive (red) phase difference feature present in all maps near the top interface. This is typically observed in areas of the film displaying a larger misalignment from the zone-axis. The line profiles of the average phase difference shown in Figure 5c confirm this trend. Following the evolution of the phase difference in the defective region (blue rectangle) as shown in Figure 5d, it can be observed that at 0.2 V the blue front first forms a bridge-like structure between dislocations. With decreasing voltage, the shape of the front changes to align with the boundary of the needle-like domain, where the ferroelectric 180° domain wall motion is hindered until the ferroelastic domain is overcome completely below −1.6 V. The trends of the line profiles in Figure 5e are very similar to those seen in Figure 5c, except for the absence of the top positive phase change present in Figure 5b. From these results, it is possible to determine the average velocity of the observed switching front and compare it with the results obtained by macroscopic approaches reported in earlier studies. In our case, the switching process occurs over the course of about 2.6 s throughout the entire 75 nm thick film. Including an error due to the finite frame time of the imaging process, we estimate the average velocity for the entire film to be about 29 nm/s. This compares well with order of magnitude estimates based on the results reported by Tybell et al. [45], who studied PZT thin films of the same composition and a similar thickness range. Furthermore, we can estimate a reduced domain wall velocity of about 22 nm/s in the defective region of the film, when compared to the predominantly defect-free region. Our observation is in agreement with previous reports that demonstrate hindering of ferroelectric 180° domain wall motion by ferroelastic *a*-domains in tetragonal PZT thin films [7]. Similarly, Gao et al. [10] reported that the pinning force of misfit dislocations on the domain wall motion slowed down the domain wall speed only slightly. 

As we show in Figure 2c and Figure 4a, slight misalignments in the specimen due to the presence of structural defects can greatly affect the DPC signal. Therefore, we need to be careful when interpreting the observed dynamic changes occurring during electrical biasing experiments as they could emerge as a result of specimen mis-tilt, induced by a thermal expansion or piezoelectric response, rather than by ferroelectric polarization switching. Typically, electrothermal expansion of the electrodes and/or the sample support result in a significant (thermal) drift and is expected to occur homogeneously throughout the film. In our experiments, however, neither of these effects are observed. Tilt-induced changes in the DPC signal emerging from the reverse piezoelectric effect also need to be addressed. In general, a change of the crystal lattice by the reverse piezoelectric effect is accompanied by changes in the ferroelectric polarization as well. In such an event, a homogeneous switching front should be observed, which is not the case in our in situ biasing experiments. Therefore, we can safely attribute the observed signal primarily to a change in the ferroelectric polarization.

## 4. Conclusions

The response of a PZT thin film ferroelectric capacitor to electrical biasing has been investigated in situ by STEM imaging. A homogeneous electric field has been purposely applied across the entire capacitor geometry and the 180° domain wall motion has been monitored by DPC-STEM imaging for the first time. Since minor sample misalignments from zone-axis result in significant phase changes in the DPC images, it is very difficult to follow the domain wall motion in the individual frames. Thus, here we propose to track the evolution of the switching front by calculating the difference between two DPC images acquired at different moments of the biasing experiment. By using this approach and overlaying the resulting switching fronts on strain maps of the PZT film we observe that the motion of the switching is hindered by both misfit dislocations and ferroelastic needle-like domains. In particular, domain wall velocities of about 29 nm/s are obtained from areas containing only misfit dislocations, while the measured velocities are decreased to 22 nm/s in regions comprising both misfit dislocations and ferroelastic *a*-domains. Aside, we observe polarization backswitching in the first 8 nm of the PZT film near the bottom interface on the removal of the applied field. Thus, opposite polarization domains in a tail-to-tail configuration are stabilized suggesting that the ferroelectric properties of the PZT films might rapidly degrade with decreasing film thickness.

While our analysis is limited by the acquisition speed of the DPC signal required to achieve a good signal-to-noise ratio, we anticipate that with the development of more sensitive pixelated direct electron detectors and the richer information provided by 4D-STEM techniques, such as the full convergent beam electron diffraction (CBED) pattern, in the future, it will be possible to resolve the ferroelectric domain wall motion in more detail. The proposed methods shall stimulate additional detailed studies on the ferroelectric switching processes and assist with the miniaturization of ferroelectric-based data storage devices.

## Data Availability

The data can be provided by authors on request.

## Supplementary Materials

The following are available online at https://www.mdpi.com/article/10.3390/ma14164749/s1, Figure S1: Topography and PFM measurement of the as-grown PZT thin film, Figure S2: (a) Atom column fitting and (b) extracted PZT lattice parameters, Video SV1: 4-segmented detector data and resulting DPC image of the in situ electrical biasing experiment.
