# Supplementary material for: Monitoring Electrical Biasing of Pb(Zr0.2Ti0.8)O3 Ferroelectric Thin Films In Situ by DPC-STEM Imaging
Source: Materials (Basel). 2021 Aug 23;14(16):4749. doi: 10.3390/ma14164749 (PMC8400982; doi:10.3390/ma14164749)
Supplement: Supplementary file 1 [file materials-14-04749-s001.zip › materials-1329985-supplementary/Supplementary Materials .pdf]

materials

MDPI

Supplementary Materials: Monitoring electrical biasing of ferroelectric Pb(Zr0.2Ti0.8)O3 thin-film in-situ by DPC-STEM imaging

Alexander Vogel [1,*], Martin F. Sarott [2], Marco Campanini [1], Morgan Trassin [2] and Marta D. Rossell [1,*]

1 Electron Microscopy Center, Empa, Swiss Federal Laboratories for Material Science and Technology, 8600 Dübendorf, Switzerland; marco.campanini1@gmail.com

2 Department of Materials, Eidgenössische Technische Hochschule Zürich, 8093 Zürich, Switzerland; martin.sarott@mat.ethz.ch (M.F.S.); morgan.trassin@mat.ethz.ch (M.T.)

* Correspondence: alexander.vogel@empa.ch (A.V.); marta.rossell@empa.ch (M.D.R.)

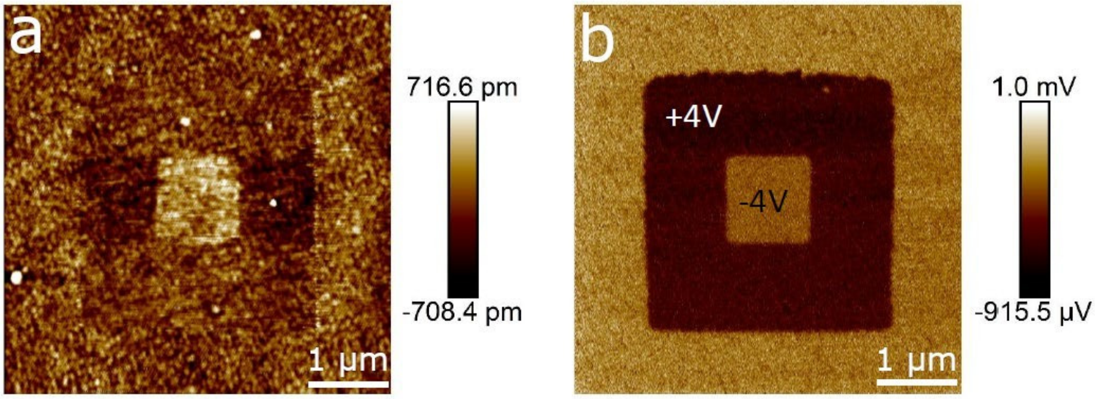

Figure S1. (a) Topography measurement performed simultaneously with the Piezoresponse force microscopy (PFM) measurement, showing a root-mean-square roughness of 0.26 nm. (b) Out-of-plane PFM measurement of the thin film. ±4 V was reversibly applied to the scanning tip to locally switch the out-of-plane oriented polarization of the film.

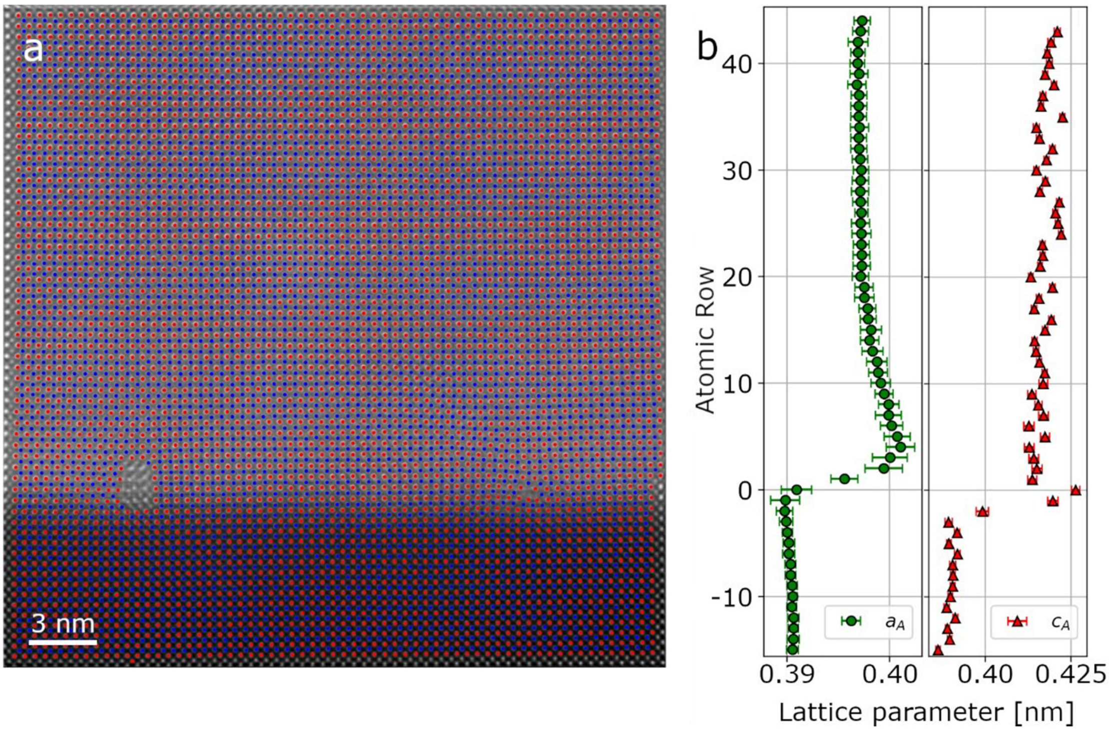

Figure S2. (a) Result of atom column fitting using atomap. (b) In-plane (a_A) and out-of-plane (c_A) lattice parameters extracted from the A-cation positions. The 0th row is chosen at the PZT/STO interface.
